# Targeting the Axl and mTOR Pathway Synergizes Immunotherapy and Chemotherapy to Butylidenephthalide in a Recurrent GBM

**DOI:** 10.1155/2022/3236058

**Published:** 2022-05-18

**Authors:** Ching-Ann Liu, Horng-Jyh Harn, Kuan-Pin Chen, Jui-Hao Lee, Shinn-Zong Lin, Tsung-Lang Chiu

**Affiliations:** ^1^Bioinnovation Center, Buddhist Tzu Chi Medical Foundation, Hualien, Taiwan; ^2^Department of Pathology, Tzu Chi University, Hualien Tzu Chi Hospital, Buddhist Tzu Chi Medical Foundation, Hualien, Taiwan; ^3^Department of Neurosurgery, Hualien Tzu Chi Hospital, Hualien, Taiwan; ^4^Tzu Chi University, Hualien, Taiwan; ^5^Everfront Biotech Inc., Taipei, Taiwan

## Abstract

**Background:**

The role of inherent tumor heterogeneity and an immunosuppressive microenvironment in therapeutic resistance has been determined to be of importance for the better management of glioblastoma multiforme (GBM). Some studies have suggested that combined drugs with divergent mechanisms may be promising in treating recurrent GBM.

**Methods:**

Intracranial sustained (Z)-*n*-butylidenephthalide [(Z)-BP] delivery through Cerebraca Wafers (CWs) to eliminate unresectable brain tumors was combined with the administration of temozolomide (TMZ), pembrolizumab, and cytokine-induced killer (CIK) cells for treating a patient with recurrent glioblastoma. Neurological adverse events and wound healing delay were monitored for estimating tolerance and efficacy. Response Assessment in Neuro-Oncology criteria were applied to evaluate progression-free survival (PFS); further, the molecular characteristics of GBM tissues were analyzed, and the underlying mechanism was investigated using primary culture.

**Results:**

Intracerebral (Z)-BP in residual tumors could not only inhibit cancer stem cells but also increase interferon gamma levels in serum, which then led to the regression of GBM and an immune-responsive microenvironment. Targeting receptor tyrosine kinases, including Axl and epidermal growth factor receptor (EGFR), and inhibiting the mechanistic target of rapamycin (mTOR) through (Z)-BP were determined to synergize CIK cells in the presence of pembrolizumab and TMZ in recurrent GBM. Therefore, this well-tolerated regimen could simultaneously block multiple cancer pathways, which allowed extended PFS and improved quality of life for 22 months.

**Conclusion:**

Given the several unique functions of (Z)-BP, greater sensitivity of chemotherapy and the synergism of pembrolizumab and CIK cells could have affected the excellent prognosis seen in this patient with recurrent GBM.

## 1. Introduction

Glioblastoma (GBM) treatment often involves aggressive surgery, radiotherapy, and chemotherapy; however, its recurrence is usually reported [1] because its highly invasive nature and rapid proliferation permit tumor cell infiltration within the functional areas of the brain, which then restricts the complete removal of the affected brain tissue. Additionally, limited treatment options for progressive/recurrent GBM typically limit lifespan to 6–9 months from the time of recurrence [2–4]. Such rapid progression of GBM is due to the proliferation of cancer stem cells, but their role in tumor heterogeneity remains unknown [5,6]. Therefore, newer and developing treatments for GBM are based on molecular and genetic profiles of cancer cells obtained from surgical specimens, wherein *ex vivo* biomarker and targeted therapeutic investigations are used to determine a precise and personalized treatment approach that can yield better outcomes.

The current standard of care, i.e., adjuvant chemotherapeutic temozolomide (TMZ) and concomitant radiotherapy (RT), or concurrent chemoradiotherapy (CCRT) after surgical tumor removal for newly diagnosed GBM, was established more than 15 years ago [1]. Among these therapeutic approaches, TMZ, an oral alkylating agent, has demonstrated antitumor activity in GBM and its recurrence [7]. However, more than 50% of patients with recurrent GBM treated with TMZ showed unsatisfactory response owing to the non-methylation of O^6^-methylguanine-DNA methyltransferase (MGMT) [8]. The other chemotherapeutic agent, carmustine (1,3-bis(2-chloroethyl)-1-nitro-sourea, BCNU), was incorporated into a controlled-release device, i.e., Gliadel™ Wafer (GW), for surgical implantation in patients with newly diagnosed and recurrent GBM [9,10]. This intracranial implant can overcome the blood-brain barrier to effectively and directly deliver cytotoxic drugs to the tumor area in the brain; however, GW-related complications or adverse reactions, including brain edema, pain (e.g., headache), and impaired wound healing, have been reported to be profoundly high and thus require careful monitoring [11].

Cytokine-induced killer (CIK) cells are characterized by the coexpression of CD3 and CD56 molecules, which have potent cytolytic activity in a MHC-unrestricted manner, both in hematological or solid malignances [12]. Moreover, CIK cells are clinically relevant as patient-derived peripheral blood mononuclear cells (PBMCs) that are easily harvested for *ex vivo* expansion and efficient production. Previous reports show that a combination of CIK cell therapy and CCRT [1] could be synergistically used for treating GBM [13], and a randomized, open-label, multi-center phase III trial, designed to assess the clinical outcomes of autologous CIK cell therapy in combination with CCRT that recruited 180 patients with GBM [14], reported median progression-free survival (PFS) of 8.1 months in the group provided CIK cell therapy combined with CCRT, compared to 5.4 months in the CCRT alone group. However, despite promising PFS, no clinical beneficial effect on the overall survival (OS) was seen.

Immunosuppressive tumors and their neighboring microenvironment in GBM have been investigated and can be partly explained by mechanisms seen in lymphopenia secondary to bone marrow suppression [15]; therefore, immunologically, GBM is a cold malignant lesion with low tumor mutation burden and few tumor infiltrating lymphocytes. Moreover, administration of steroids may be necessary for the management of brain edema in patients with high-grade gliomas; this leads to reduced efficacy when using immunotherapies [16]. Interaction between the programmed cell death protein 1 (PD-1) and its ligand (PD-L1) could create an immunoregulatory axis that promotes the invasiveness of GBM tumor cells [17] because PD-L1 expression on the tumor surface activates PD-1 receptor in the microglia, leading to a negative regulation of T cell responses [18]. *Cancer* cells are also found to induce PD-L1 secretion by activating various receptors [19], such as Toll-like receptor (TLR) [20], epidermal growth factor receptor (EGFR) [21], interferon alpha receptor (IFNAR) [22], and interferon gamma receptor (IFNGR) [23], and while a blockade of PD-1/PD-L1 immune checkpoints has yielded significant improvements in several kinds of tumors [24,25], it is not so with GBM. Recently, two phase III studies evaluated the efficacy of anti-PD-1 antibody using a different regimen. Specifically, one study used a combination of surgical tumor removal and adjuvant pembrolizumab, with or without neoadjuvant pembrolizumab administration [26], and demonstrated a surprisingly long median OS of 13.7 months ((7 days) in the neoadjuvant pembrolizumab group, compared to 7.5 months (228 days) in the control group. In contrast, efficacy data from the other clinical trial that used neoadjuvant nivolumab reported a median OS of 7.3 months [27], and the differences were found between the two studies.

Management strategies for GBM can be multidisciplinary combinations, including those that inhibit the proliferation of cancer stem cells, downregulate the expression of MGMT to resensitize cells to TMZ, convert an immunosuppressive cold microenvironment to a hot one, and activate exogenous immune cells after systemic administration of a promising drug candidate. Therefore, a locally delivered polymer containing a newly characterized active pharmaceutical ingredient, (Z)-*n*-butylidenephthalide [(Z)-BP], was designed and produced as a Cerebraca Wafer (CW) to treat glioma [28–30] by directly implanting it into the surgical cavity created when a brain tumor is resected. Cerebraca Wafer is an investigational product of Everfront Biotech Inc. The excipient of the Cerebraca Wafer is a biodegradable polyanhydride CPPSA copolymer (similar to that in the Gliadel Wafer) that is biocompatible and has been intracranially implanted to animals and patients for more than decades. The CW (300 mg) is composed of poly[carboxyphenoxy-propane/sebacic acid] anhydride containing 25% (Z)-BP (75 mg) and has drug retention for over 14–21 days after being placed onto the surface of the resected tumor cavity in the brain. (Z)-BP, discovered in a traditional Chinese medicinal plant, *Angelica sinensis* [31], can be chemically synthesized and owes its antitumor activity, including the inhibition of cancer stem cell and reduction of metastasis capabilities, to Axl targeting [29,30]. Given that the downstream mTOR pathway inhibition can be associated with (Z)-BP-inactivated receptor tyrosine kinase including Axl [32,33], several cancer pathways are blocked [34,35], and a synergistic effect with TMZ is then achieved [36,37].

Here, we describe the case of one patient with GBM recurrence who was provided compassionate use of a six-piece CW implantation, adjuvant TMZ oral medication, CIK cell infusion, and pembrolizumab (immune checkpoint inhibitor) administration. This combination was well-tolerated, and neither abnormal wound healing nor brain edema was observed. A positive clinical response, i.e., PFS, has continued for more than 22 months after the treatment, and the patient remained under follow-up till August 2021.

## 2. Materials and Methods

### 2.1. Medical History of the Patient and the Study Design

**Figure 1:**
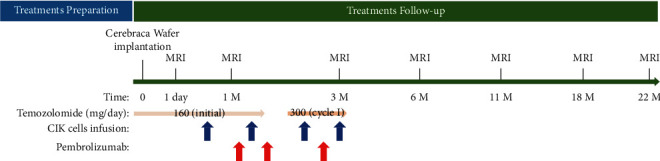
Overview of the treatment regimen. After the patient underwent surgical implantation of six CWs on day 0, TMZ administration, in the initial phase (days 1–50) and in cycle I of the maintenance phase (days 79–84), was dispensed to the patient for use according to instructions. Infusion of CIK cells was performed on days 19, 40, 61, and 82, post-surgery. Between TMZ and delivery of CIK cells, the patient was also provided intravenous injections of pembrolizumab on days 29, 50, and 71. T1-weighted MRI with gadolinium enhancement (∼22M) and T2-fluid attenuated inversion recovery (FLAIR) (∼3M) of the patient's brain at the indicated schedule.

A 43-year-old male had previously undergone surgery for grade II glioma (World Health Organization, WHO classification) in the right parietal region, and 2 years after the first diagnosis, a follow-up magnetic resonance imaging (MRI) of the brain showed evidence of tumor recurrence. Thus, a second surgery for the removal of a tumor that was pathologically diagnosed as GBM (Supplemental Figure 1) was performed, with concomitant use of RT and adjuvant TMZ. Nine months later, GBM progression was managed using gamma knife radiosurgery, followed by bevacizumab administration. Thirty-one months from the date of the second craniotomy, T1-weighted MR images with a contrast agent showed evidence of recurrent tumors. Given the limited therapeutic options for GBM, the patient was provided compassionate use of the newly developed CW (Everfront Biotech Inc., New Taipei City, Taiwan) and adjuvant TMZ. This compassionate use of CWs for recurrent GBM was approved by the Research Ethics Committee of the Hualien Tzu Chi Hospital (SP108-03). This third tumor debulking surgery was guided using fluorescein sodium [38] under microscope (Leica M530 OHX), and the six pieces of CWs were used to cover the cavity created by tumor removal. On day 19 after CW implantation, CIK cells (1 × 10^9^) were intravenously injected—in total, four such injections were provided with a 20-day interval between injections. One month after brain surgery, 200 mg of pembrolizumab was administered as an intravenous injection at 3-week intervals for a total of three cycles (Figure 1). Disease progression was determined using the Response Assessment in Neuro-Oncology (RANO) criteria [39].

### 2.2. Patient-Derived Primary Culture Cells

Brain tumor tissue was obtained from the patient after he provided informed consent. Patient-derived primary culture cells were isolated using a protocol approved by the Research Ethics Committee of Hualien Tzu Chi Hospital (IRB106-57-A). Briefly, the samples were dissociated to a single cell suspension using a papain dissociation system (LK003150, Worthington Biochemical Corporation, Lakewood, NJ, USA) as per manufacturer's instructions as follows: Tissue solution was collected in calcium- and magnesium-free Hank's balanced salt solution and digested with papain (20 units/ml papain) in Earle's balanced salt solution (EBSS) with shaking for 1.5 hr at 37°C. After enzyme digestion, the cell-containing solution was passed through a 40-*μ*m cell strainer (BD, San Jose, CA, USA), followed by centrifugation at 300 × *g* for 5 minutes, and the pellet was resuspended in EBSS containing DNase I (100 units/ml) and albumin ovomucoid protease inhibitor (1 mg/ml). Next, a discontinuous density gradient was carefully prepared and layered using the albumin ovomucoid protease inhibitor to purify the primary culture cells. Harvested cells were resuspended at a density of 4 × 10^6^ cells/mL in DMEM/F12 medium containing 10% fetal bovine serum (FBS, HyClone, GE Healthcare Life Science, South Logan, UT, USA) and cultured in a chamber with 5% CO_2_ at 37°C and constant humidity. GBM spheroids were cultured in Neurobasal medium (NB medium) supplied with 1x B27, N2 supplement, 10 ng/mL human bFGF, and 10 ng/mL hrEGF (human recombinant EGF) in the meantime.

### 2.3. Molecular Characterization of GBM Tissues

Surgical specimens from the patient were fixed and prepared for GBM subtyping. Briefly, paraffin sections were subjected to a programmed procedure for the removal of paraffin and rehydration through xylene and a graded ethanol series. Antigen retrieval was performed, followed by incubation in 3% hydrogen peroxide to quench endogenous peroxidase activity. Tissue sections were incubated in 5% bovine serum albumin for 1 h at room temperature to limit non-specific binding; primary antibodies including epidermal growth factor receptor (EGFR, 05–104, Merck Millipore, Billerica, MA, USA) and its variant (EGFR*vIII*) (Ab00184-L8A4, Absolute antibody, Cleveland, UK), Axl (HPA037423, Sigma-Aldrich, St. Louis, MO, USA), Nur77 (Ab217547, Abcam, Cambridge, UK), and IDH1^R132H^ (DIA-H09, Dianova, Hamburg, Germany) were probed and incubated at 4 overnight [5]; specific secondary HRP-conjugated antibodies were used as needed, and DAB staining, as per manufacturer's protocols, was used for visualization. All tissue sections were counterstained with hematoxylin and mounted, and digital photos were taken using an IX70 (Olympus, Tokyo, Japan) microscope equipped with a CCD.

To assess the methylation status of MGMT (O^6^-methylguanine-DNA methyltransferase) promoter, tumor samples were collected and prepared for CpG region analysis from 3 to 7 (YGTTTTGYGTTTYGAYGTTYGTAGG). PyroMark Q24 MGMT kit (Qiagen, Hilden, Germany) was used to detect the level (%) of methylation at positions 17–39 in exon 1 of *MGMT* gene, which contains five CpGs.

### 2.4. Western Blotting Analysis

Protein lysates of primary culture cells isolated from the GBM were resolved on sodium dodecyl sulfate-polyacrylamide gels (SDS-PAGEs) and electro-transferred to polyvinylidene fluoride (PVDF) membrane (Merck Millipore) in a wet blot format. After protein transfer, the membranes were blocked with blocking buffer (5% non-fat dry milk powder, dissolved in 0.1% PBS-Tween 20) and incubated with primary antibodies against MGMT (2739S, Cell Signaling, Beverly, MA, USA), EGFR (GTX628887, GeneTex, Hsinchu, Taiwan), EGFR (phosphor Tyr1068) (GTX132810, GeneTex, Hsinchu, Taiwan), Axl (H-124) (sc-20741, Santa Cruz Biotechnology, Dallas, TX, USA), phospho-Axl (Y779) (MAB6965, R&D Systems, Minneapolis, MN, USA), mTOR (AB32028, Abcam), phospho-mTOR (Ser2448) (5536S, Cell Signaling), PD-L1 (14-5982-85, Thermo Fisher Scientific, Waltham, MA, USA), or *β*-actin (A5441, Sigma-Aldrich) that were diluted in blocking buffer and incubated overnight at 4°C. Next, the membranes were washed with 0.1% PBS-T; the HRP-conjugated secondary antibody was diluted in blocking buffer and incubated for 1h at room temperature. Enhanced chemiluminescence reagents were used to evaluate protein levels using the image detection system LAS-3000 (Fujifilm, Tokyo, Japan).

### 2.5. Cell Survival Analysis

GBM cells isolated from patient tissue were seeded at a density of 4 × 10^3^ cells/well in 96-well plates, treated with vehicle control, (Z)-BP (Everfront Biotech Inc.) was incubated alone, or in combination with 3-methyl-(triazen-1-yl)imidazole-4-carboxamide (MTIC; the active species of TMZ; M760000, Toronto Research Chemicals, Ontario, Canada), MTIC alone, or BCNU (C0400, Sigma-Aldrich) for 24 hours, followed by cell viability assessment. The CCK-8 cytotoxicity assay (MedChemExpress, Monmouth Junction, NJ, USA), a colorimetric alternative to radioactive cytotoxicity evaluation, was used. Aliquots from all tests were transferred to another 96-well flat clear bottom plate, mixed with CCK-8 reagents, and incubated for 120 minutes at 37°C with protection from light. Absorbance was recorded within 1 hour of adding stop solution, and cell viability was measured spectrophotometrically (Multiskan Go, Thermo Fisher Scientific) at a wavelength of 450 nm. All experiments were performed thrice with six technical replications.

### 2.6. Flow Cytometry Analysis

Primary cultured GBM speroid cells were seeded at 5 × 10^5^ cells per ultra-low attached TC culture dish and incubated at 37°C in humidified 5% CO_2_ for 24 hours. Next, trypsinized cells were stained with PE-CD133 antibody (130-113-108, Miltenyi Biotec, Bergisch Gladbach, Germany), or APC-SOX2 antibody (IC2018 A, R&D systems, Minneapolis, MN, USA) for 60 minutes at room temperature. Control groups were unstained samples, or those incubated with PE-conjugated anti-human IgG Fc (410707, BioLegend), with or without compensation beads (01-3333-42, Thermo Fisher Scientific). A gated positive signal corresponding to PE-CD133 or APC-SOX2 antibodies was used with compensation beads. The cell sample was analyzed using flow cytometry (CytoFlex flow cytometer, Beckman Coulter, Indianapolis, IN, USA).

### 2.7. Preparation of Activated Immune Cells and Interferon Gamma Quantification

Preparation of activated immune cells and interferon gamma quantification is described in Supplementary Materials.

## 3. Results

### 3.1. Well-Tolerated Combination of Cerebraca Wafers and Other Treatments Can Improve the Patient's Prognosis

The patient provided informed consent to undergo fluorescence-guided brain tumor resection and surgical implantation of six pieces of CWs. After the wound healed, he was prescribed TMZ capsules, and intravenous delivery of pembrolizumab and autologous CIK cells was scheduled (Figure 1).

**Figure 2:**
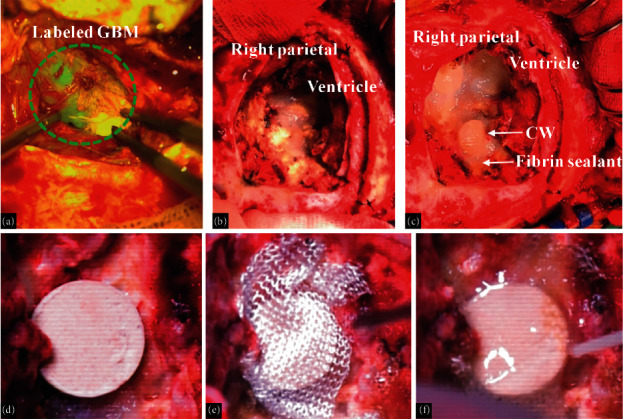
Intracranial procedure combining sodium fluorescein-guided tumor resection and Cerebraca Wafer implantation. (a) Glioma cells (green fluorescence) were visualized using a yellow (560-nm wavelength) light and resected as extensively as possible. (b) The ventricle of the right parietal was sealed with absorbable biomaterials. (c) The suspected residual tumor cavity was covered with CWs. (d) Near view of CW. (e) CW fixed with bioabsorbable hemostatic meshes. (f) Fibrin sealant materials on hemostatic meshes.

**Figure 3:**
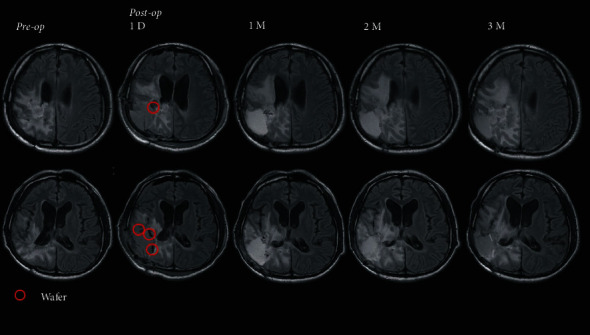
Clinical T2-FLAIR MRI showing brain edema surrounding the right parietal surgical site after intracranial implantation of Cerebraca Wafers. Axial MR T2 FLAIR demonstrating brain edema on the right parietal region. After CWs were implanted (red circles) at suspected residual tumors, stability of brain edema in the 3-month follow-up period, and ventricle size could be observed without significant changes (lower panels).

Fluorescein sodium was used during surgery to improve the rate of gross total resection and visualize the tumor [38,40] (Figure 2(a)). Next, six pieces of CWs were placed into the suspected residual tumor cavity and fixed with bioabsorbable hemostatic meshes and fibrin sealant (Figures 2(b)–2(f)). During the initial 3-month follow-up period, there were no obvious changes in brain edema, as indicated by T2-weighted fluid attenuated inversion recovery (T2 FLAIR) imaging analysis (Figure 3). No common wound complications or CW-related adverse reactions were recorded, and this combined treatment was well-tolerated.

**Figure 4:**
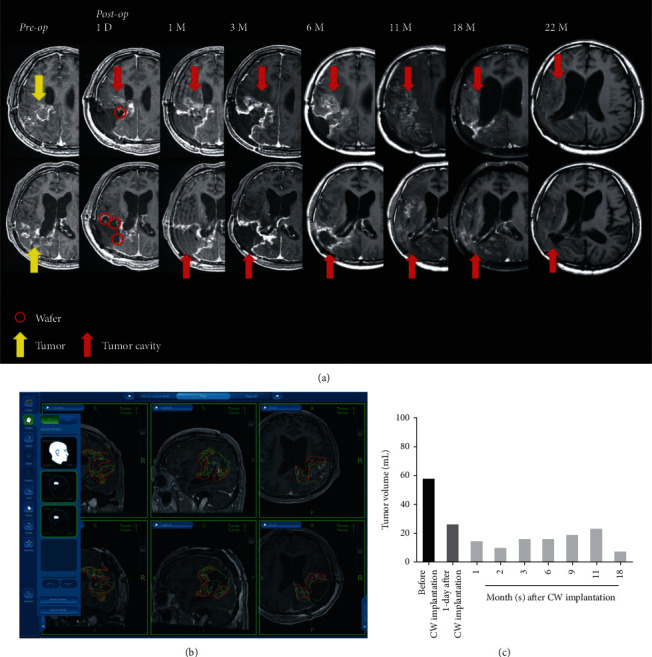
Clinical local control after intracranial implantation of Cerebraca Wafers, medication with TMZ, and infusion of pembrolizumab and CIK cells. (a) Axial MRI (T1-weighted with gadolinium enhancement) of the right parietal brain highlighting the cavity of tumor resection where CWs covered suspected residual tumors (red circles indicated CWs and yellow arrows marked resected tumors). During follow-up at 22 months with MRI, these enhancements were stable or had decreased without multifocal GBM (red arrows labeled tumor cavity). (b) Tumor volumes were calculated using regions of interest (ROI) in green line that correspond to pre-operative tumors and the red lines indicate residual malignant parts. (c) Extent of resection was ∼55% at day 1 of post-procedure, and the tumor volumes gradually regressed during 22 months after CW implantation.

The clinical trial of CW implantation and adjuvant TMZ has been published previously, which is a single-arm, open-level, 3 + 3 dose-escalation phase I clinical trial with a total of 12 patients with recurrent GBM included. Those patients receive 1 to 6 wafers implantation (according to their assigned cohort) after surgical resection of brain tumor. TMZ was administered at 75 mg/m^2^/day for 42 days and a further 200 mg/m^2^/day for 5 days every 4 weeks thereafter. No CW-related adverse events (AEs) or serious AEs (SAEs) were reported in this clinical trial. The median OS of patients receiving high-dose CW has exceeded 17.4 months, and a 100% PFS rate at six months was achieved [41]. Given that the safety profile of CW implantation and adjuvant TMZ has been reported, its drug response was analyzed using T1-weighted MR images with contrast (Figure 4(a)). The enhanced area (yellow arrows) indicates invasive GBM recurrence prior to surgery. In T1/T2-weighted images, several red circles mark a hyperintense rim around the hypointense center exhibited by the implanted CWs on day 1 post-surgery; CWs are not so clearly seen in subsequent images. During the 22 months of follow-up, contrast enhancement was noted to be stable, and the tumor cavity did not show obvious progression. Tumor volumes were calculated (Figures 4(b), 4(c)) based on contracted T1-weighted MR images using a navigation system software (Stealth Station S7, Medtronic, Minneapolis, MN, USA), and the histograms of tumor volume showed regression from 57.8 ml prior to surgery to 26.1 ml on day 1 (55% resection rate) and to 7.5 ml at 18 months, after intensive combined treatments. Additionally, the Karnofsky Performance Status (KPS) score of the patient improved from 40 points before surgery to 70 points at 6–18 months post-procedure (Supplemental Figure 2). Taken together, the combination of surgical implantation of CWs, adjuvant TMZ, and immunotherapy/cell therapy was well-tolerated, could significantly extend PFS, and improve prognosis.

### 3.2. Intermediate Methylated MGMT Promoter and Highly Expressed GFAP/Ki67 Were Identified in the GBM Tissues

**Figure 5:**
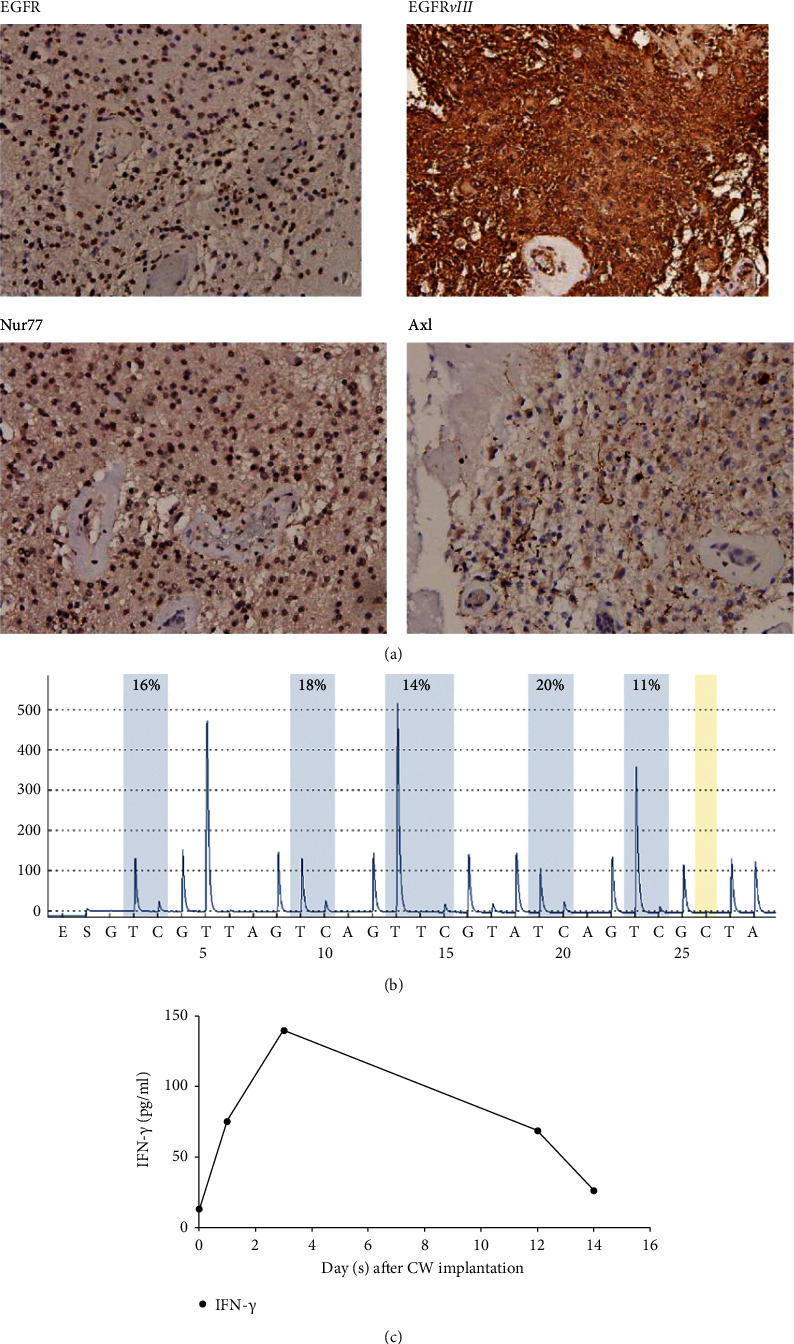
Molecular characterization of patient-derived GBM samples. (a) Representative H&E stain and immuno-histochemistry results of molecular markers showed the expression of GFAP, and Ki67 and IDH1 R132H were probed. (b) MGMT promoter methylation status was determined by measuring the percentage of methylated MGMT promoter in these five indicated CpG regions. Mean range of the regions <10%,10–26%, and ≧27% was considered as unmehtylated, intermediate methylated, and menthylated, respectively. (c) Detection of IFN*γ* in the patient's serum from pre-operative day 0 to day 14 post-surgery. Baseline values on day 0 were 13.25 pg per milliliter, and the increase in levels of IFN*γ* appeared to correspond with CWs implantation. After 3 days, IFN*γ* levels started to decline in a time-dependent manner, which correlate with the slow release of (Z)-BP.

Patient-derived surgical specimens were subjected to molecular analysis, which revealed the tissue to be GBM with the tumor cells highly expressing GFAP, Ki67, and IDH1 R132H (Figure 5(a)). Tumor DNA was extracted from these samples, and pyrosequencing analysis revealed that 5 CpG regions of the MGMT promoter were intermediate methylated (Figure 5(b)), which could be indicated by the average percentage of methylated CpG site in the mean range of 10–26%. Reference [42]. Such data can be one of the prognosis tools or clinical predictors and may reflect response to TMZ and OS [8].

### 3.3. Local and Controlled Release of (Z)-BP Inhibited Axl and mTOR Activity to Synergize Immunotherapy for Cancer Stem Cell Elimination

Implantation of CWs resulted in a rapid tenfold increase in IFN*γ* levels from 13.25 pg/mL before surgery to a maximum of 140.31 pg/mL on day 3. Further, the slow controlled-release wafers delivering (Z)-BP led to a sustained twofold increase in IFN*γ* during 2 weeks after surgery (Figure 5(c)). An *in vitro* cytotoxic assay was also used to reveal if abundant IFN*γ* secreted by activated commercial PBMCs occurred due to (Z)-BP exposure (Supplemental Figure 3), and we found that high serum IFN*γ* levels did not affect neurologic function or the general status of the patient (Supplemental Figure 2), and that such levels might convert the cold tumor microenvironment to a “hot” one that is responsive to the synergistic action of PD-L1 inhibitor (pembrolizumab) administration.

**Figure 6:**
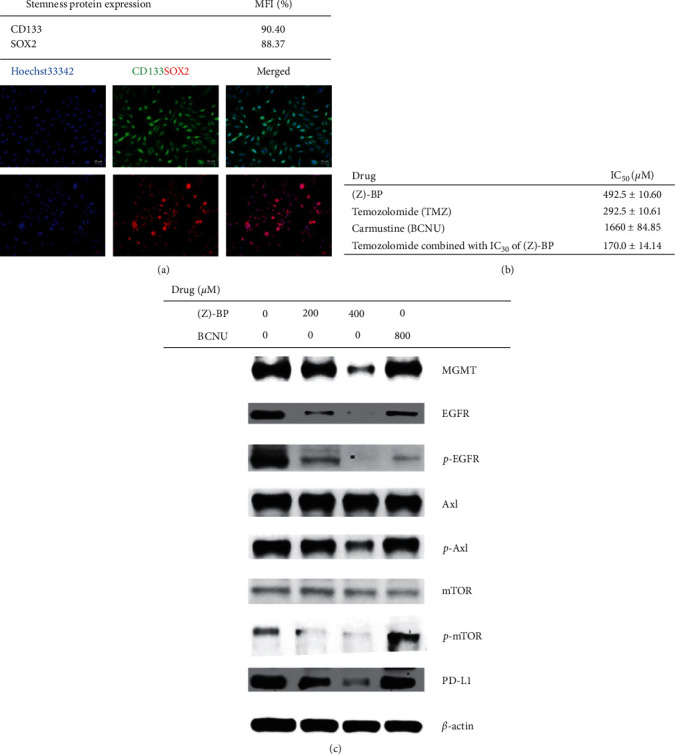
Clinical drug responses were evaluated in the patient-derived primary culture GBM cells. (a) Most of the primary culture cancer cells were CD133 (green) and SOX2 (red), highly expressed using flow cytometry analysis and immunofluorescence assay. Hoechst33342-labeled nuclei. Bar = 50 *μ*m. MFI: mean fluorescent intensity. (b) Flow cytometry analysis and immunofluorescence assay in GBM primary cultured spheroid cells to detect the high expression of CD133 (green) and SOX2 (red) cultured with serum-free medium. Hoechst33342-labeled nuclei. Bar = 50 *μ*m. MFI: mean fluorescent intensity. (c) Primary culture cancer cells, treated with (Z)-BP and temozolomide (active form MTIC was used), presented relatively low IC_50_, compared to BCNU. Additionally, dosing (Z)-BP in IC_30_ concentration could reduce the required amount of TMZ for determining the synergistic effect on eliminating primary culture gliomas. (d) Using (Z)-BP (200 or 400 *μ*M) to expose the primary culture cells could reduce the level of MGMT, in comparison to vehicle control and BCNU (800 *μ*M). Further, receptor tyrosine kinases including EGFR, phosphorylated EGFR, and Axl were also downregulated. Upstream mTOR activity was inactivated (p-mTOR) by (Z)-BP, and this could mutually affect Axl and EGFR signaling and then decrease the protein level of PD-L1. *β*-Actin was used as an internal control.

Patient-derived primary culture cells, consisting of abundant cancer stem cells expressing CD133 and SOX2, were acquired and analyzed (Figure 6(a)), and the effects of treatment with carmustine (BCNU) or (Z)-BP, in the presence/absence of TMZ, were evaluated (Figure 6(b)). Compared to the active pharmaceutical ingredient in GW, i.e., BCNU, the IC_50_ of (Z)-BP was significantly lower; notably, a lower concentration of (Z)-BP used (one-third to one-fourth of BCNU) could effectively inhibit the proliferation of patient-derived glioblastoma cells. Additionally, compared to TMZ alone, a combination of (Z)-BP and TMZ indicated that much lower doses of TMZ could effectively suppress up to 50% of GBM cells in primary culture. Western blotting analysis of MGMT expression in primary culture cancer cells showed that the inhibitory ability of (Z)-BP on MGMT could result in TMZ re-sensitization (Figure 6(c)). Such high expression of MGMT levels was also consistent with the results of pyrosequencing data (Figure 5(b)). Together, these results indicate that (Z)-BP enhances the response to TMZ in patient-derived primary culture by downregulating the expression of the DNA repair enzyme MGMT.

Next, to investigate the action of (Z)-BP in glioma, the receptor tyrosine kinase pathway was examined (Figure 6(c)). Western blotting analysis showed the downregulation of EGFR and phosphorylated Axl and concurrent reduction in activated mTOR. These findings and the increase in IFN*γ* concentration, both *in vitro* and *in vivo*, indicated that the investigation of PD-L1 signaling was necessary [43,44], and we found that (Z)-BP could reduce the expression level of PD-L1 in patient-derived primary culture cells. Thus, targeting Axl and thereby inhibiting downstream mTOR signaling can reduce PD-L1 protein expression, and our results suggest optimal use of rational combinations to synergize the clinical efficacy of immunotherapies.

## 4. Discussion

The patient described in this report participated in a compassionate use study, and a phase IIa study (NCT03234595) to evaluate the efficacy of CWs with adjuvant TMZ therapy in patients with high-grade glioma is currently underway. CWs displayed excellent local control in our patient with recurrent GBM, along with improvements in KPS and PFS (more than 22 months), indicating that CIK cell therapy and pembrolizumab immunotherapy could enhance systemic responses. Overall, our clinical experience in a phase I study of CW combined with TMZ administration and expanded access to add-on immune therapies against glioma provide initial evidence on the safety profile and antitumor response of CW therapy.

We report that (Z)-BP released from CWs targeted Axl [29,30], which is a receptor tyrosine kinase involved in tumor growth and metastasis and an indicator of poor prognosis in several cancers including GBM [45], and that it performed well to extend the PFS in a patient with recurrent GBM. Significant improvements in quality of life and KPS scale were also seen for more than 22 months. Recurrence is common even in patients with fluorescence-guided gross total resection (>90%) of brain malignancies [46], and relapsed tumors occur within a 2-cm range of the resected tumor margin [47]. Although the GBM resection rate in this case was 55%, the observed clinical response could be attributed to the local and controlled-release of (Z)-BP in high concentrations and a combination of TMZ, pembrolizumab, and CIK cell therapies. Loading drugs in a wafer formulation, i.e., CW (25% (Z)-BP), has resulted in greater drug abundance than GW (3.85% BCNU) with the added advantage of an approximate 67% reduction in IC_50_ of (Z)-BP, which is significantly lower than that of BCNU. The patient-derived cancer stem cells abundantly expressed CD133 and SOX2, which are related to recurrence [48]; importantly, these molecules were effectively inhibited by (Z)-BP during cancer cell proliferation, implying that high concentrations of (Z)-BP could increase the distance of drug penetration into residual tumors or other brain areas, compared with GW [29], thereby controlling disease recurrence (Figure 4). These positive results apart, challenges associated with the use of CWs for treating high-grade gliomas include wafer migration-induced obstructive hydrocephalus [49], tumor heterogeneity, and the immunosuppressive microenvironment [5,15]; nevertheless, this study provides proof-of-concept results that confirm (Z)-BP to be a promising small molecule that can be used in therapy against high-grade gliomas, and that combination therapy can synergize antitumor activity against a devastating cancer type. After brain tumor debulking [40], hemostatic matrix agents were used to prevent bleeding.

*Cancer* therapy is a hard and tough challenge for scientists and doctors due to the tumor diversity and variable cancer behaviors during or after the treatment. Therefore, precisely personalized medicine can be one of the standard criteria for treating malignancy including glioblastoma and organoid-based ex vivo model. The decision of therapeutic approaches is including the consideration of multidisciplinary treatments, such as the combination of butylidenephthalide (API of CW) and Temozolomide (TMZ) in our study, which have been emphasized on the characters of patient-derived samples. This efficacy can be predicted and evaluated by *ex vivo* examinations, and meticulous treatment combination may be planned. CWs were placed at suspected residual/unresectable tumor tissues and secured to the tumor cavity using bioabsorbable meshes and biomaterials to avoid fluid flushing and rapid metabolism of (Z)-BP. Although a fibrin sealant can prevent hydrocephalus due to wafer migration, desired local high concentrations of (Z)-BP might not be achieved.

This case highlights the potential of localized high-dose (Z)-BP with respect to antitumor responses and indicates that doses of (Z)-BP and excipient of up to 450 (75 × 6) mg and 1,350 (225 × 6) mg, respectively, are tolerated. During the first 3 months post-surgery, CW-related adverse drug reactions were not noted on physical examination, KPS score, electrocardiography (EKG), MRI, or laboratory testing, including hematology and biochemistry. Notably, neither brain edema nor hydrocephalus was observed. This is in contrast to results from our previous toxicological study of CW in animals, where a few animals experienced increased intracranial pressure, brain edema, and hemorrhage, but no significant systemic adverse reactions. Further, management in the intensive care unit with intracranial pressure monitoring as the standard of care after craniotomy also reduced the possibility of infection and other complications.

This case highlights the utility of combined treatment regimens in eliciting favorable clinical responses in one patient with a classical type of GBM intermediate methylated MGMT promoter. The treatment of high-grade glioma or GBM with a single therapeutic agent can be difficult [1] as this type of tumor requires a multi-pronged approach that involves surgery, drug, cell, and targeted therapies. Due to individual differences among patients and tissue heterogeneity within gliomas, precise and personalized therapeutic methods are needed to obtain better outcomes. In this case, the tissue tested negative for several biomarkers of GBM classification, including neurofibromatosis (NF1), TP53, platelet-derived growth factor receptor alpha polypeptide (PDGFRA), and neurofilament light chain (NEFL), indicating a classical subtype of GBM favored. While intracranial CW implantation appeared to control local tumor recurrence, TMZ administration was used to synergistically resensitize mTOR suppression in order to modulate MGMT protein expression. Thus, two cycles of oral TMZ medication with drug resistance could effectively enhance the systemic response and decrease new lesion recurrence.

To understand the mechanisms of (Z)-BP action, patient-derived primary culture cells were used to evaluate potential drug targets. Previous reports have shown that inhibition of Axl and EGFR dimerization result in mTOR inactivation [33], and our data with (Z)-BP-induced inactivation of Axl concur, with the mechanisms being decreased phosphorylation and downregulation of EGFR expression. Additionally, inactivation of Axl or mTOR contributing to the downregulation of PD-L1 and modulation of IFN*γ* production have been described [50], which are consistent with our results. Thus, CWs could reverse an immunosuppressive tumor microenvironment by increasing IFN*γ* production and inhibiting PD-L1 expression, which then probably activated autologous CIK that synergized (Z)-BP and acted on the GBM. Compared to the systemic administration of immune checkpoint inhibitors, intracerebral administration of (Z)-BP could not only increase local therapeutic concentration and decrease the dose level of TMZ, but also reduce systemic immune-related adverse events [51]. IFN*γ* upregulation implicates the alteration of glioma microenvironment. The poorly immunogenic or “cold” lesion of tumors contains cytotoxic lymphocytes (CTLs) with low expression of IFN*γ* and poor infiltration of CTLs into the tumor core, which are immunological ignorance. In contrast, the immunologically “hot” tumors are termed Infiltrated-inflamed (I–I) tumor immune microenvironment with increased expression of IFN*γ*. These results in the manuscript suggest that the implantation of Cerebraca Wafer alters the glioma microenvironment due to the increase in IFN*γ* expression. The interferon-*γ* (IFN*γ*) analysis of patient serum confirms this. In cocultures with immune cells, (Z)-BP administration increased IFN*γ* expression levels 1.63-fold, indicating a positive antitumor immune response. Taking together, our study states briefly that (Z)-BP contributes to immune modulation.

CCRT is the standard of care in the treatment of high-grade glioma; however, tumors usually recur within 12–18 months, and newly developed therapeutic options involving checkpoint inhibitors such as PD-1 and PD-L1 have been associated with minimal improvement in OS and limited efficacy [27]. Several checkpoint inhibitors of T cells, including cytotoxic T lymphocyte-associated antigen-4 (CTLA-4), lymphocyte activation gene-3 (LAG3), indoleamine 2, 3 dioxygenase (IDO), 4-1BB (CD137), and OX40 (CD134), which are effective in inhibiting T cell function, should also be considered. Compared to clinically applicable GWs, (Z)-BP in CW can not only reduce nuclear receptor subfamily 4 group A member 1 (Nur77) to increase cancer cell apoptosis [35], but also inhibit telomerase reverse transcriptase (TERT) to promote cell senescence [28]. Tissue from the patient in this report was characterized based on these biomarkers; hence, (Z)-BP wafer implantation, combined with TMZ and immunotherapy, was recommended.

Although we demonstrated an exciting result of Cerebraca Wafer treatment in this study, however, limitations exist which would be addressed. First, Cerebraca Wafers (CWs) directly cover and come in contact with the cavity wall created by tumor removal. Therefore, the CW might be limited to use to treat GBM present in the brainstem or basal skull lesions which are difficult to approach implantation. Furthermore, the pharmacokinetic of CW is around 28 days after implantation in the resected tumor cavity in the brain. To eradicate residual or inaccessible tumor, oral or nasal administration of BP (API of Cerebraca Wafer) would be applied in the future. Our study reveals that the quantity of BP obtained from the brain tissue by nasal administration was approximately 10-fold higher than the quantity obtained from the oral-administrated brain tissue samples [52]. Notably, the development of nanoparticles for nasal administration of BP along with Cerebrca Wafer implantation is a perspective in GBM therapy.

## 5. Conclusion

The report describes encouraging data on one patient, but CW is still a study drug with adjuvant TMZ in the clinical investigation for efficacy evaluation. Safety profiles of CW have been addressed in a phase I study and recently published in Cancers and a phase II study for efficacy is ongoing. So far, the clinical experience using CW to treat glioblastoma is accumulating and more than twenty patients with brain tumor are implanted with one-to-six pieces of wafers. However, the limitation of this case report can be only one patient receiving concurrent CWs implantation and immunotherapies. Additionally, of the twenty subjects treated, only six were the classical subtype of glioma. A complicated tumor composition and heterogeneous types may contribute to different results of the clinical responses. Therefore, the precision medicine of CW application for the suitable patient population will be addressed, and the risks of using this investigation product are also carefully considered. Targeting Axl signaling may be one criterion applied to patients expressing activated Axl proteins, and the results of this case report can suggest the optimal use of rational combinations to synergize the clinical efficacy of immunotherapies.

## Data Availability

The data presented in this study are available in the article.

## Supplementary Materials

Supplementary Materials The supplementary materials include preparation of activated immune cells and interferon gamma quantification (supplementary figures 1, 2, and 3).
