# Supplementary material for: Targeting the Axl and mTOR Pathway Synergizes Immunotherapy and Chemotherapy to Butylidenephthalide in a Recurrent GBM
Source: J Oncol. 2022 May 18;2022:3236058. doi: 10.1155/2022/3236058 (PMC9132698; doi:10.1155/2022/3236058)
Supplement: Supplementary Materials — The supplementary materials include preparation of activated immune cells and interferon gamma quantification (supplementary figures 1, 2, and 3). [file 3236058.f1.zip › 3236058.f1/2022 5 2 Supplemental materials.docx]

**Supplemental materials**

*Preparation of activated immune cells and interferon gamma quantification*

Peripheral blood mononuclear cells (PBMCs) were obtained from ATCC (PCS-800-011™) and cultured in DMEM/F12 with 10 % FBS (11039021, Thermo Fisher Scientific). Briefly, PBMCs were seeded at a density of 1 × 10^6^ cells/ml for cell expansion. The medium contained an equal number of Dynabeads Human T-activator CD3/CD28 (11161D, Thermo Fisher Scientific), 20 IU/ml recombinant human interleukin-2 (rhIL-2, Novartis, Basel, Sweden), and 20 ng/ml anti-CD3 monoclonal antibody (Takara, Mountain View, CA, USA). These cells were incubated at 37℃ in an atmosphere with 5 % CO_2_ for 5 days, followed by (Z)-BP treatment for 24 hours. IFNγ was quantified using an enzyme-linked immunosorbent assay kit (BGK01579, BioGems, Westlake Village, CA, USA) that was pre-coated with human IFNγ biotinylated antibody. Presence of biotinylated antibody was detected enzymatically using streptavidin-HRP. A cutoff value of 100 pg/mL was defined as a positive cell response and was set beforehand. The assay-sensitivity level was 20–1000 pg/mL.

**Supplemental figures**

Supplemental Figure 1. Histology and pathology of brain tumor progression in the patient.

Tissue section with the use of hematoxylin and eosin (HE) staining of (A) low grade glioma in the first operation was showed. After that, the disease was progressed and diagnosed as secondary GBM (B). Standard cares including brain tumor removal and chemo-radiotherapy were applied to treating GBM disease, but these malignant glioma cells remained to be proliferated and accordingly leaded to the recurrence (C). Scale bar=50 μm.

Supplemental Figure 2. Karnofsky Performance Status score of the patient with GBM was improved.

Prior to the surgical removal of tumor and CWs implantation, the patient needed supports from family and scored with 40-point. After surgical combination of CWs and different treatments, the KPS scale was largely improved to 70 points at 6-month of post operation. Till now, patient’s performance remains maintained.

Supplemental Figure 3. Level of IFNγ was increased by treating GBM cancer stem cell (1XM) and stimulated commercial PBMCs with (Z)-BP.

While applying activated PBMCs to culture with the GBM cancer stem cell line (1XM), derived from the radiotherapy-resistant primary culture glioma, detection of IFNγ was determined using ELISA. Data indicated a significant difference between control and (Z)-BP (400 μM) treatment. ***: *p* < 0.001 (Student’s *t*-test).
