# Supplementary figures and images for: Targeting the Axl and mTOR Pathway Synergizes Immunotherapy and Chemotherapy to Butylidenephthalide in a Recurrent GBM
Source: J Oncol. 2022 May 18;2022:3236058. doi: 10.1155/2022/3236058 (PMC9132698; doi:10.1155/2022/3236058)

## Slide 1
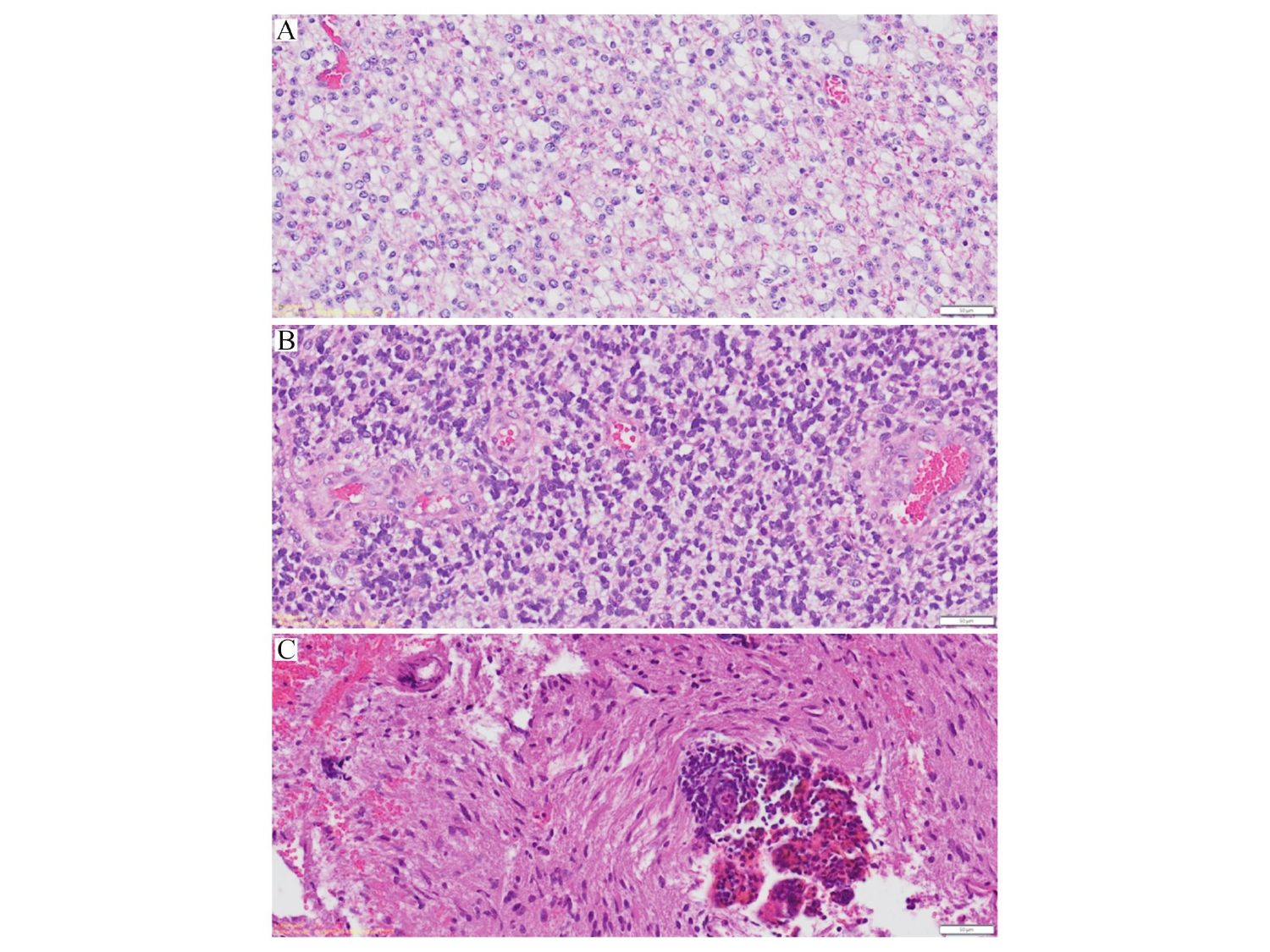

Supplement: Supplementary Materials — The supplementary materials include preparation of activated immune cells and interferon gamma quantification (supplementary figures 1, 2, and 3). [file 3236058.f1.zip › 3236058.f1/Supplemental Figure 1.pptx]

## Slide 1
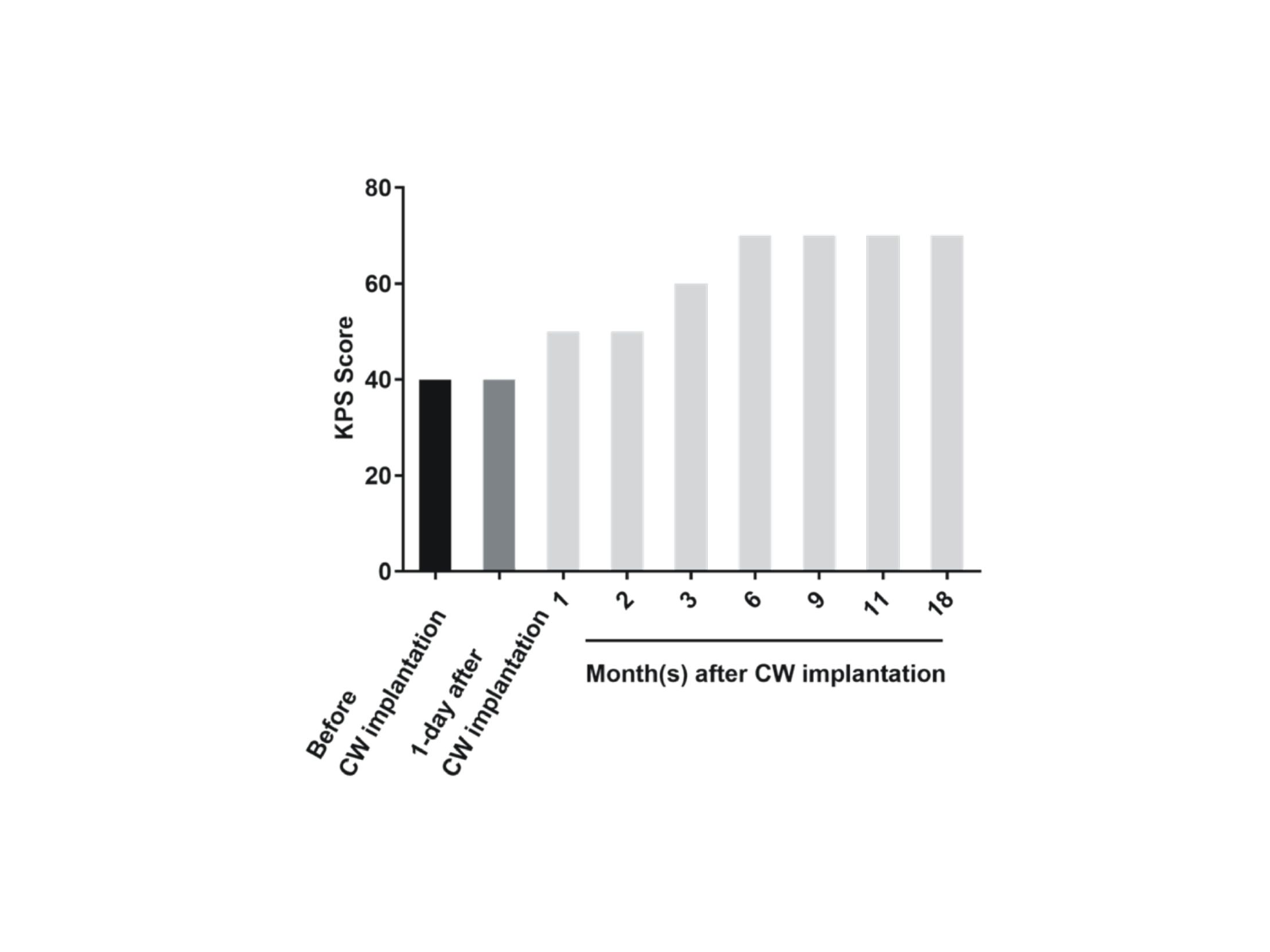

Supplement: Supplementary Materials — The supplementary materials include preparation of activated immune cells and interferon gamma quantification (supplementary figures 1, 2, and 3). [file 3236058.f1.zip › 3236058.f1/Supplemental Figure 2.pptx]

## Slide 1
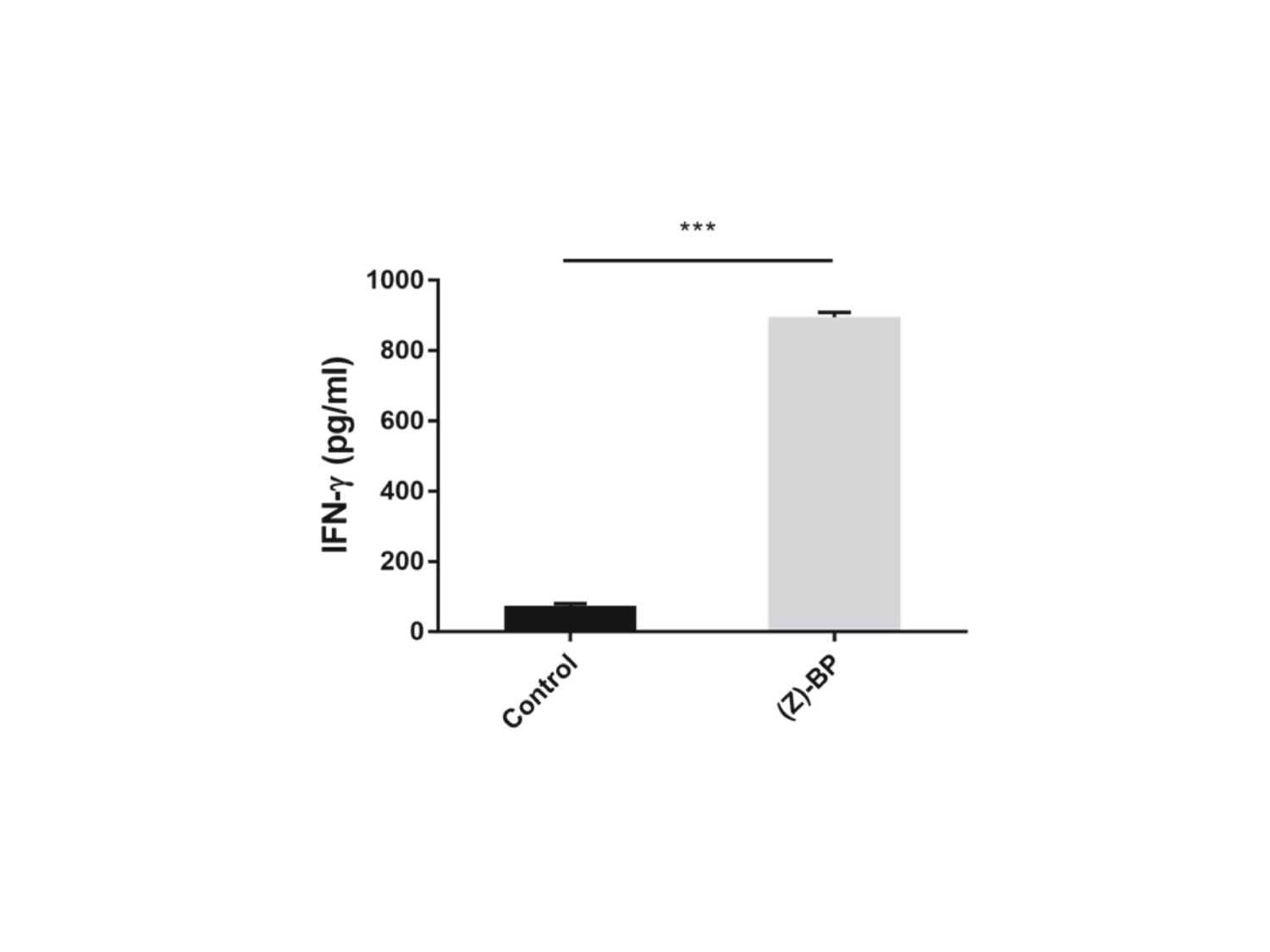

Supplement: Supplementary Materials — The supplementary materials include preparation of activated immune cells and interferon gamma quantification (supplementary figures 1, 2, and 3). [file 3236058.f1.zip › 3236058.f1/Supplemental Figure 3.pptx]
